# Enhancing Multiple Ways of Knowing: Comment on "Evaluating Public Participation in a Deliberative Dialogue: A Single Case Study"

**DOI:** 10.34172/ijhpm.2023.7776

**Published:** 2023-04-15

**Authors:** Katherine M. Boydell

**Affiliations:** ^1^Black Dog Institute, Sydney, NSW, Australia; ^2^Department of Psychiatry and Mental Health, Faculty of Medicine, University of New South Wales, Sydney, NSW, Australia; ^3^Dalla Lana School of Public Health, University of Toronto, Toronto, ON, Canada

**Keywords:** Deliberative Dialogue, Policy-Making, Public Participation, Knowledge Translation Strategy

## Abstract

This commentary reviews the Scurr and colleagues’ article published in *International Journal of Health Policy and Management* in February 2022 on "Evaluating Public Participation in a Deliberative Dialogue: A Single Case Study." Schur adds to the current knowledge base by extending the stakeholder groups in deliberative dialogues (DD) to members of the affected community, a practice not commonly used in such DD strategies. Their study supports the inclusion of public participants in such dialogues, and offers practical guidelines for ways in which to accommodate these important participants. This commentary highlights the need to acknowledge diverse types of knowing into what is considered evidence and advocates for evidence to include a wide-ranging variety of sources including tacit knowledge via experience and ongoing learning.

 Deliberative dialogues (DDs) are increasingly being used, particularly as a knowledge translation (KT) strategy.^1-6^ They are a promising interactive KT approach that brings together and influences the knowledge of diverse stakeholders who are critical resolving a societal issue. The process involves a purposeful, facilitated discussion about a pressing issue that needs to be addressed and with the goal of identifying an agreed-upon path of action. Our own work in using DDs in the mental health and suicide prevention field has demonstrated that the bringing together of key stakeholders and leaders strengthens debate and knowledge, harnesses action around shared purpose and strengthens collaboration.^7,8^ Like others, we note how critical it is for professional and lived experience stakeholders to have forthright and open conversations about power differentials and the overt transfer of power if we are to address the current rhetoric about the value of co-led and co-designed projects.

 Scurr and colleagues’ article^9^ adds to the extant [and relatively sparse] knowledge base that supports the inclusion of pubic participants in DDs, an evidence-based KT strategy. Scurr et al extend this literature and address a current gap in research by providing an in-depth exploration of the impact of community participation in the DD process. They demonstrate that involving key stakeholder groups in such dialogues brings different and valued perspectives and experiences to policy discussions.

 Scurr et al emphasise the multiple ways of knowing that inform the DD; formal scientific knowledge, professional knowledge and local knowledge [or citizen evidence]. This is a critical point and aligns well with previous research that has facilitated our understanding of the significance of different evidence bases, and especially of the need to include healthcare service users and public citizens in decision-making.^10^ In particular, is the work by Gabbay and Le May^11,12^ who highlight that patients and health service users generate valid knowledge and can be viewed as ‘experts.’ With the current emphasis on person-based medicine, it is critical to challenge conventional evidence-based medicine, which tends to view knowledge narrowly and often excludes experiential wisdom.^11^

 Scurr and colleagues found that the civic participants [tenants in rent-geared-to-income housing] expressed their tacit knowledge in the discussion via narratives — personal and detailed experiential stories. In order to enhance the development of trust, they advocate for allowing tenants the space to discuss their challenges and feel that their voices are heard. They found that professional participants failed to share their stories as frequently or extensively as civic participants, which is expected given the goal of offering a safe space to share narratives (taking care to ensure they did not veer into airing of grievances) Scurr et al suggest that this could be encouraged in future DDs. In addition to valuing experiential wisdom is the need to acknowledge the challenges to involvement of civic participants in such dialogues which include time and effort that require acknowledgement. In addition, mentoring support and training should underpin any project. DDs are not aimed at achieving consensus, so it is interesting to note that tenants in the study highly valued achieving consensus whilst the professional stakeholders did not.

 Patients, healthcare services users and, in the case study by Scurr et al, tenants of a rent-geared-to-income housing project, have a right to have input to research on their experiences. Decreasing the extant power imbalances between researchers and study participants is critical, particularly with marginalised and seldom-heard groups. In the Scurr article, power dynamics featured largely in the planning process as well as in the dialogues themselves. Oliver and Pearce also reflect on the central issue of power with respect to balancing researcher, clinician and public knowledge without privileging some experiences and perspectives over others.^13^ They note the need for application of research evidence in a wide variety of settings and with a balance with other interests, with the possibility of extending the use of deliberative processes to cultivate consensus between distinctive stakeholder values and priorities. They argue that it is fundamental to recognise the role of power in evidence-informed decision-making and the corresponding need to examine who wields it and how. Oliver and Pearce conclude that, rather than acknowledging that power imbalances exist, underlying power imbalances in the production and use of knowledge must be described, understood and addressed. Research teams are encouraged to adopt a process of critical reflection and dialogue throughout every project, constantly considering power sharing and their own positionality vis-à-vis others.^14^

 In summary, the work of Scurr and colleagues is vital and has important analytic generalisability, that is, the ability to extend the findings beyond the current study to a larger population or to differing contexts beyond that which was studied. This piece of work is extremely relevant to the burgeoning literature on best practice in co-production and co-creation projects.^15,16^

## Ethical issues

 Not applicable.

## Competing interests

 Author declares that she has no competing interests.
